# Urinary Metabolic Profiling in Volunteers Undergoing Malaria Challenge in Gabon

**DOI:** 10.3390/metabo12121224

**Published:** 2022-12-06

**Authors:** Madeleine Eunice Betouke Ongwe, Isabelle Kohler, Mikhael D. Manurung, Aswin Verhoeven, Rico Derks, Jacqueline J. Janse, Yoanne D. Mouwenda, Peter G. Kremsner, Ayola A. Adegnika, Bertrand Lell, Bart Everts, Oleg A. Mayboroda, Maria Yazdanbakhsh

**Affiliations:** 1 Department of Parasitology, Leiden University Medical Center, 2333 ZA Leiden, The Netherlands; 2 Centre de Recherches Médicales de Lambaréné, Lambaréné P.O. Box 242, Gabon; 3 Institut de Recherches en Écologie Tropicale, Centre National de la Recherche Scientifique et Technologique (CENAREST), Libreville P.O. Box 13354, Gabon; 4 Division of BioAnalytical Chemistry, Amsterdam Institute of Molecular and Life Sciences (AIMMS), Vrije Universiteit Amsterdam, 1081 HV Amsterdam, The Netherlands; 5 Center for Analytical Sciences Amsterdam, 1081 HV Amsterdam, The Netherlands; 6 Center for Proteomics and Metabolomics, Leiden University Medical Center, 2333 ZA Leiden, The Netherlands; 7 Institut für Tropenmedizin, Eberhard-Karls-Universität Tübingen, 72074 Tübingen, Germany; 8 German Center for Infection Research (DZIF), 38124 Braunschweig, Germany; 9 Department of Medicine I, Division of Infectious Diseases and Tropical Medicine, Medical University of Vienna, 1090 Vienna, Austria

**Keywords:** urine, metabolomics, *Plasmodium falciparum* malaria, nuclear magnetic resonance, hydrophilic interaction chromatography-mass spectrometry, Gabon

## Abstract

The interaction of malaria parasites with their human host is extensively studied, yet only few studies reported how *P. falciparum* infection affects urinary metabolite profiles and how this is associated with immunity. We present a longitudinal study of the urinary metabolic profiles of twenty healthy Africans with lifelong exposure to malaria and five malaria-naïve Europeans, who were all challenged with direct venous inoculation of live *P. falciparum* sporozoïtes (PfSPZ) and followed up until they developed symptoms or became thick blood smear positive (TBS). Urine samples were collected before and at 2, 5, 9 and 11 days post challenge and were analysed. Upon infection, all Europeans became TBS positive, while Africans showed either a delay in time to parasitaemia or controlled infection. Our metabolic data showed that Europeans and Africans had distinct alterations in metabolite patterns, with changes mostly seen on days 5 and 9 post PfSPZ infection, and more prominently in Europeans. Within the African group, the levels of formate, urea, trimethylamine, threonine, choline, myo-inositol and acetate were significantly higher in TBS positive whereas the levels of pyruvate, 3-methylhistidine and dimethylglycine were significantly lower in individuals who remained TBS negative. Notably, before inoculation with PfSPZ, a group of metabolites including phenylacetylglutamine can potentially be used to predict parasitaemia control among Africans. Taken together, this study highlights the difference in urinary metabolic changes in response to malaria infection as a consequence of lifelong exposure to malaria and that change detectable before challenge might predict the control of parasitaemia in malaria-endemic areas.

## 1. Introduction

Human malaria caused by *Plasmodium falciparum* remains a major public health problem, particularly in sub-Saharan Africa. Artemisinin-based combination therapies are the current treatment. However, resistance to this treatment has been emerging [1,2,3], which stimulates the current search for the development of new and effective treatments and/or vaccines. For this, a better understanding of the molecular mechanisms underlying host–parasite interaction is needed. The interaction of malaria parasites with their human host has been studied [4,5] using untargeted and targeted metabolomics [6,7,8,9]. The use of metabolomics for studying malaria was pioneered by Ghosh and Sengupta [10,11,12,13], who first used murine models and later characterized the plasma metabolite fingerprints in severe malaria infected individuals [14]. Most of metabolomics studies that have investigated human malaria have been cross-sectional, and performed on either serum or plasma samples, identifying altered metabolites associated with malaria infection chronicity [15] or severity [16]. To our knowledge, only one study has investigated the urinary metabolite profiles, which was performed in an acute malaria case-control study and identified certain metabolites such as pipecolic acid, taurine, N-acetylspermidine, N-acetylputrescine and 1,3-diacetylpropane as biomarkers of *P. falciparum* infection in adult Ethiopians [17].

Compared with blood, urine is a fluid that is easy to collect in a non-invasive fashion and is available in large volumes. Urine is composed of breakdown products of endogenous and exogenous metabolite sources, reflecting the physiological state of an organism at a certain time point. As such, urinary metabolite composition and levels reflect changes in the all-body metabolism and organ function. Little is known about the metabolic response to *P. falciparum* infection before the development of parasitaemia and symptoms. Controlled Human Malaria Infection (CHMI) provides a great opportunity to investigate host–parasite interactions. It consists of the administration of live sporozoites to healthy volunteers, either by injection via a needle or bites of infected mosquitoes, under controlled settings where study participants are closely followed up to assess the development of parasitaemia and symptoms [18]. We have used midstream urine samples from a CHMI trial conducted in Gabon where 20 Africans volunteers with lifelong exposure to malaria parasites and 5 malaria-naïve Europeans were inoculated with live, aseptic, cryopreserved *P. falciparum* sporozoites (PfSPZ) [19]. While all Europeans developed early parasitaemia after receiving direct venous administration of sporozoites, the Africans showed better control of parasitaemia, and a subset of individuals remained free of parasites as detected by microscopic examination of thick blood smear (TBS) indicating naturally acquired immunity (NAI) against malaria. Therefore, this CHMI can provide a unique approach to investigate the early perturbations of the urinary metabolome after *P. falciparum* infection and assess whether any profiles are associated with the control of parasitaemia.

## 2. Material and Methods

### 2.1. Study Design and Sample Collection

**Figure 1:**
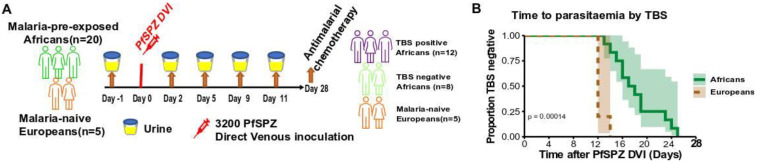
Study design. (**A**) A total of 25 healthy volunteers, 20 malaria-exposed Africans and 5 malaria-naive Europeans, received direct intravenous administration of 3200 live, aseptic, cryopreserved *P. falciparum* sporozoïtes (PfSPZ DVI). (**B**) Survival curves showing the time to parasitaemia defined by thick blood smear (TBS) microscopy until Day 28 when antimalarial chemotherapy was given in Europeans and Africans. The log-rank test was used to compare the survival distributions of the two groups.

This study took place in Lambaréné, Gabon, where 20 malaria-pre-exposed African volunteers with lifelong history of *P. falciparum* exposure and five malaria-naïve Europeans without any history of malaria exposure were enrolled in CHMI trial. The trial is registered with ClinicalTrials.gov, number NCT02237586 and was approved by the Gabonese National Ethics Committee. The study was conducted in accordance with the declaration of Helsinki. Before the trial started, all volunteers gave their written consent. The five malaria-naïve volunteers had no resided in the study area for an average of 5 months and had no previous malaria history whereas lifelong malaria exposed Africans volunteers were resident of the study area and had experiences with malaria. Before CHMI, all included volunteers started a regimen of 300mg of clindamycin for 5 days, a highly efficacious and well-tolerated antimalarial drug with a short half-life of 2–4 h, that targets all parasites stages without consequences on CHMI trial. Two days after antimalarial treatment, all volunteers were challenged by direct venous inoculation (DVI) of 3200 live, aseptic, cryopreserved *P. falciparum* sporozoites (PfSPZ) and were closely monitored throughout the trial for 28 days. Anti-malarial treatment was administered when participants had a thick blood smear (TBS) positive and had malaria-associated symptoms or a parasitaemia above 1000 µL. All volunteers without parasitaemia or remaining at low density parasitaemia without symptoms were treated on Day 28. The details of the study, characteristics of the volunteers (Table S1), selection criteria and trial outcomes are published elsewhere [19]. The morning midstream urine samples were collected from the volunteers at Day 1 before PfSPZ DVI, as well as on Days 2, 5, 9, and 11 post-PfSPZ DVI (Figure 1A). After collection, urine samples were directly put on ice first, then aliquoted in 1.5 mL cryotubes and immediately frozen at −80 °C at the Centre de Recherches Médicales de Lambaréné (CERMEL) in Gabon and were transported on dry ice to Leiden for metabolomic analysis.

### 2.2. Targeted Analysis Using Nuclear Magnetic Resonance Spectroscopy

The urine samples were randomised and organised in the acquisition blocks of 96 samples. Prior acquisition, urine samples were thawed and 700 μL of each urine sample was transferred to a 96 deep-well plate and centrifuged at 3000 rpm for 5 min. Of each sample, 630 μL of each sample was mixed with 70 μL of potassium phosphate buffer (1.5 M, pH 7.4) in 100% D2O containing 4 mM TSP and 2 mM NaN3 and transferred to a 5 mm Bruker Sample Jet NMR tubes. Proton nuclear magnetic resonance spectroscopy (^1^H NMR) data was obtained using a Bruker 600 MHz AVANCE II spectrometer equipped with a 5 mm TCI cryogenic probe head and a z-gradient system. A Bruker Sample jet changer was used for sample insertion and removal. A fresh sample of 99.8% methanol-d_4_ was used for temperature calibration before each batch of measurements. One-dimensional (1D) ^1^H NMR spectra were recorded at 300 K using the first increment of a NOESY pulse sequence with pre-saturation (γB_1_ = 50 Hz) during a relaxation delay of 4 s and a mixing time of 10 ms for efficient water suppression [20]. Initial shimming was performed using the Topshim (Bruker, 2011) tool on a random mix of urine samples from the study, and subsequently the axial shims were optimized automatically before each measurement. Sixteen scans of 65,536 points covering 12,335 Hz were recorded and zero-filled to 65,536 complex points prior to Fourier transformation. An exponential window function was applied with a line-broadening factor of 1.0 Hz. The spectra were automatically phased, and baseline corrected and automatically referenced to the internal standard (TSP = 0.0 ppm). Duration of 90° pulses were automatically calibrated for each individual sample using a homonuclear-gated mutation experiment [21] on the locked and shimmed samples after automatic tuning and matching of the probe head. J-resolved spectra (JRES) were recorded with a relaxation delay of 2 s and 2 scans for each increment in the indirect dimension. A data matrix of 40 × 12,288 data points was collected covering a sweep width of 78 × 10,000 Hz. A sine-shaped window function was applied, and the data was zero-filled to 256 × 16,384 complex data points prior to Fourier transformation. In order to remove the skewing, the resulting data matrix was tilted along the rows by shifting each row (k) by 0.4992*(128-k) points and symmetrised about the central horizontal lines. For the targeted analysis, the raw data were exported into the Chenomx NMR suite 9.0 software (Chenomx Inc.) where 53 metabolites were annotated and quantified using a batch fit tool and manual curation. Concentrations (mM) were extracted using the known TSP concentration (0.4 mM) (Table S2).

### 2.3. Untargeted Analysis Using Hydrophilic Interaction Chromatography—Mass Spectrometry

Urine samples were defrosted at room temperature and 4-fold diluted with a mixture of acetonitrile (MeCN)/water 75:25 (*v/v*). The prepared samples were randomized prior to the analysis by hydrophilic interaction chromatography—mass spectrometry (HILIC-MS), which also involved the analysis of quality control samples (QCs), consisting of a standard solution of metabolites covering a large range of physico-chemical properties and eluted over the whole chromatogram range, as well as quality matrix samples (QC pool), consisting of a pool of the 96 samples analysed the first day of experiments. QCs and QC pool samples were repeatedly injected over the day-to-day sequences and used to evaluate and ensure the data reliability, including intra- and inter-day repeatability of retention times and peak areas. HILIC experiments were carried out using a Dionex Ultimate 3000 LC system (Thermo Scientific/Dionex, Amsterdam, The Netherlands) equipped with a solvent degasser, a column oven, a thermostatic autosampler and a Dual Gradient Rapid Separation pump allowing for parallel LC analysis. Exploratory experiments were performed with a Luna HILIC (i.e., Diol chemistry) column from Phenomenex (Utrecht, The Netherlands) of 100 × 2.00 mm i.d., 3 µm. The mobile phase was composed of (1) 50 mM ammonium acetate buffer adjusted with acetic acid at pH 6.0 and (2) MeCN. The flow rate was set to 600 µL/min with the following gradient profile: 95% B for 1 min, 95–35% in 3.25 min, 35% B for 0.75 min, and 95% for 2 min for a total analysis time of 7 min. Two columns from the same batch were used in parallel, allowing for a simultaneous equilibration of one column (95% B) during the sample analysis occurring in the second column. Samples were thermostatic at 4 °C and analyses carried out at 40 °C. A HILIC Security Guard cartridge from Phenomenex of 4 × 2.00 mm i.d., 3 µm, was used as a pre-column. Chromeleon software version 6.80 SR12 (Thermo Scientific/Dionex) was used for LC control. The LC system was hyphenated using a 1:10 splitter to a Maxi’s impact UHR-q-tof mass analyser (Bruker Daltonics, Bremen, Germany) via an electrospray ionization (ESI) source operating in negative ionization mode over the range 50 to 1000 *m/z* with an acquisition rate of 1 Hz. ESI capillary voltage and end plate offset were set at 4000 V and 500 V, respectively. Drying gas flow rate and temperature were fixed at 6 L/min and 200 °C, respectively, and nebulizing gas pressure at 1.5 psi. A hydro-organic solution of H_2_O/*i*-PrOH 50:50 (*v/v*) containing sodium formate clusters was infused at the beginning of each analysis to allow for mass recalibration. HyStar version 3.2 was used for ESI-q-tof/MS control, data acquisition, and data handling. We obtained 385 features (Table S3). All MS data files were recalibrated based on sodium formate *m/z* clusters. The LC-MS data files were exported in *mzxml* format and aligned with the in-house developed alignment algorithm msAlign2 available on www.ms-utils.org/msalign2, accessed on 15 February 2015. Peak picking was performed with XCMS package (The Scripp Research Institute, La Jolla, CA, USA) based on the *centWave* algorithm using the following settings: maximal tolerated *m/z* deviation in consecutive scans, 5 ppm; peak width, 5–40 s; scan range to process, 36–360; noise, 10,000; prefilter step, at least 3 peaks with intensity > 10,000; *m/z* centre of the feature, *wMean* (intensity weighted mean of the feature *m/z* values); signal-to-noise ratio threshold, 20; minimum difference in *m/z* for peaks with overlapping migration time, 0.001 min; integration method, peak limits found through descent on the real data. The Probabilistic Quotient Normalization method was used to account for the dilution of the samples [22]. Water and acetonitrile (MeCN) were obtained from Sigma-Aldrich (Schnelldorf, Germany). Ammonium acetate and sodium hydroxide were purchased at Sigma-Aldrich. Formic and acetic acid were obtained from Biosolve (Valkenswaard, The Netherlands). Data were log transformed and mean centred.

### 2.4. Data Analysis and STATISTICS

Statistical analyses of metabolite concentrations obtained using NMR were performed using the R statistical software version 4.0.2. To analyze differences in metabolite abundances per time point, we performed linear mixed-model analysis on log2-transformed values with the following fixed effects: group (malaria-naïve or malaria-exposed; parasitaemic (TBS positive) and non-parasitaemic (TBS negative), time (coded as a categorical variable), an interaction term between group and time, sickle-cell trait, and sex. Patient ID was included as the random intercept term. We used distinct variance parameters at each time point for each group using the *varIdent* function and autoregressive (AR1) correlation structure. The model was fit using the restricted maximum likelihood method using the *lme* function from the *nlme* R package. To test for differences between groups within time points or within groups between time points, a post-hoc contrast analysis was coded as appropriate using the *emmeans* R package. To test for the interaction effect between time and group, *joint_tests* function from the *emmeans* R package was used. Data wrangling and visualisation was performed using the *tidyverse* R package. To explore for the most discriminating metabolites between Europeans and Africans at baseline (Day 1 before PfSPZ DVI), we performed sparse partial least-squares discriminant analysis (sPLS-DA) from mixOmics R/Bioconductor package (version 6.14.1). The number of features and selection of distance measures were tuned using k-fold cross-validation with 1000 repeats. Upon the selection of parameters for the final sPLS-DA model, the stability of features was assessed using k-fold cross-validation with 1000 repeats. For mass spectrometry data, the features contributing to the sPLS-DA model were ranked using the variable important for projection (VIP) values. In order to identify the metabolites of interest, chemical formulae were generated based on the internally calibrated monoisotopic mass within 5 ppm mass error and submitted to the METLIN Metabolite Search (http://metlin.scripps.edu) and the Human Metabolome Database (HMDB, http://www.hmdb.ca), both accessed on 25 May 2015. The confirmation of the identity of metabolites of interest was carried out with the injection of neat standards via comparison of retention time, as well as MS and MS/MS data. All statistically significant comparisons are annotated using asterisks * *p* < 0.05, ** *p* < 0.01, *** *p* < 0.001.

## 3. Results

### 3.1. Comparison of Urinary Metabolites in Europeans and Africans upon Plasmodium falciparum Infection

As indicated in Figure 1A, 20 Africans with lifelong exposure to malaria and 5 malaria naïve Europeans were infected with PfSPZ by direct venous injection (DVI). As already published [19], after PfSPZ DVI, all 5 Europeans (100%) and 12 of 20 Africans (60%) developed parasitaemia detectable by thick blood smear microscopy (TBS positive), while 8 remained negative (TBS negative) throughout the 28-days study period (Figure 1B and Table S1).

**Figure 2:**
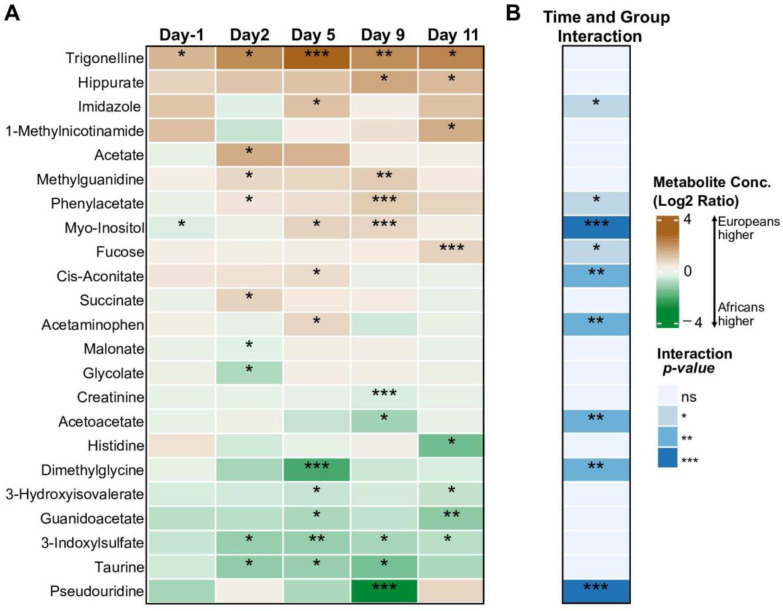
Changes in urinary metabolites in Europeans and Africans upon Plasmodium falciparum infection. (**A**) Heatmap showing differentially abundant urinary metabolites between Europeans and Africans on Day-1 (before PfSPZ DVI), and at 2, 5, 9, and 11 days post PfSPZ DVI. Color indicates Log_2_-ratio of metabolite levels between Europeans and Africans; positive values indicate higher metabolite concentrations in Europeans. (**B**) Metabolites with differential trajectory of change between the groups in response to the challenge (time and group interaction effect). Statistically significant comparisons are indicated using colors and asterisks, as shown in the legend. * *p* < 0.05, ** *p* < 0.01, *** *p* < 0. 001.

A total of 53 metabolites were identified and quantified in urine samples using the targeted NMR approach (Table S2). Analysing the metabolite concentrations at different time points, 23 metabolites were significantly different between Europeans and Africans (both TBS positive and TBS negative together), at any given time point (Figure 2A). One metabolite trigonelline was elevated in Europeans at all times points. Metabolites that showed significant differential changes over time between Europeans and Africans are given in Figure 2B. These metabolites are imidazole, phenylacetate, myo-inositol, fucose, cis-aconitate, acetaminophen, acetoacetate, dimethylglycine, and pseudouridine. As shown in Figure S1, the changes over time were largely seen in European volunteers, while metabolite concentrations in Africans did not seem to get perturbed much following malaria infection. The strongest changes were observed at Days 5 and 9 post PfSPZ DVI (Figure 2A). Overall, the longitudinal data showed that the urinary concentrations of a number of metabolites detected by NMR were differentially altered during the progression of *P. falciparum* infection particularly in the malaria naïve Europeans.

### 3.2. Comparison of Urinary Metabolites in TBS Positive and TBS Negative AFRICANS after PfSPZ DVI

**Figure 3:**
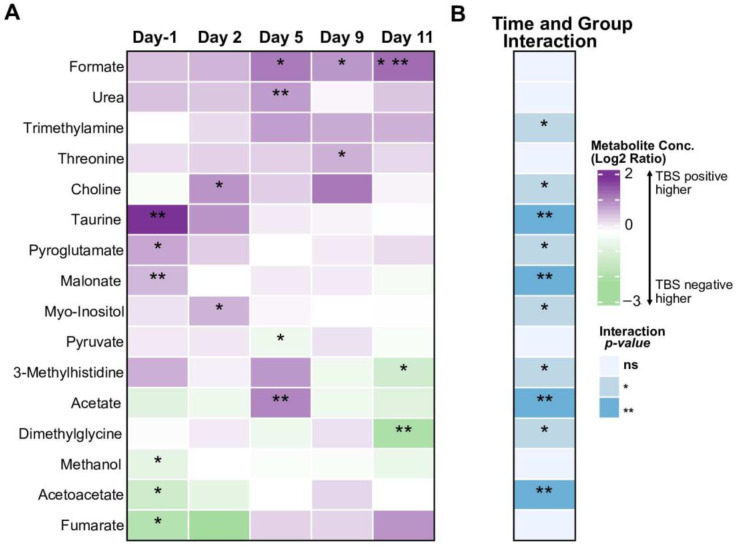
Comparison of urinary metabolites in TBS positive and TBS negative Africans after PfSPZ DVI. (**A**) Heatmap showing differentially abundant urinary metabolites between TBS positive and TBS negative Africans on Day-1 (before PfSPZ DVI), and at 2, 5, 9, and 11 days post PfSPZ DVI. Color indicates Log_2_-ratio of metabolite levels between the two groups; positive values indicate higher metabolite concentrations in TBS positive Africans. (**B**) Metabolites with differential trajectory of change between the groups in response to the challenge (time and group interaction effect). Statistically significant comparisons are indicated using colors and asterisks, as shown in the legend. * *p* < 0.05, ** *p* < 0.01.

In malaria-exposed Africans, 40% remained TBS negative throughout the 28 days of the study period, which indicates the ability to control parasitaemia. We thus compared urinary metabolite concentrations over time between TBS positive and TBS negative Africans (Figure 3A). Metabolites that showed significant differential changes over time between TBS positive and TBS negative Africans are given in Figure 3B and Figure S2. These metabolites were trimethylamine, choline, taurine, pyroglutamate, malonate, myo-inositol, 3-Methylhistidine, acetate, dimethylglycine and acetoacetate. Two of the metabolites, namely myo-inositol and dimethylglycine, that over time changed differently between Europeans and Africans (Figure 2), were also seen to change differently between TBS positive and TBS negative Africans.

### 3.3. The Differences in Urinary Metabolites at Baseline

**Figure 4:**
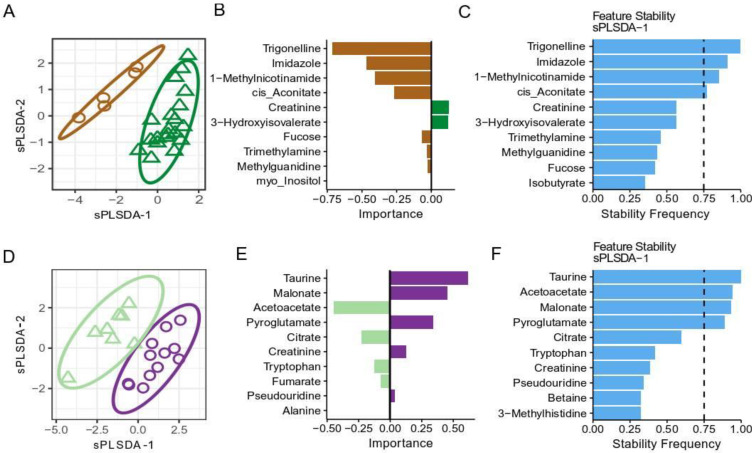
The differences in urinary metabolites at baseline. Sparse PLS-DA (sPLS-DA) analysis of metabolite concentrations at baseline (at Day 1 before PfSPZ DVI) in (**A**) Europeans (brown color) and Africans (green color) and as well as in (**D**) TBS negative (light green color) and TBS positive (purple color) Africans. The following hyperparameters were used for both sPLS-DA models in (**A**,**D**): two components, maximum distance, 10 variables per component. Model classification performance was assessed using 5-fold cross-validation. Overall classification error rates for the first and second component for (**A**) were 28% and 29%, respectively, and for (**D**) were 25% and 21%, respectively. (**B**,**E**) Importance of metabolites in the first sPLS-DA component for (**A**), and (**B**), respectively. Color indicates the group in which the metabolite concentration is higher. (**C**,**F**) represented the frequencies in which the metabolites were retained over 1000 repeats of 5-fold cross-validation, indicating the reproducibility of the signature.

Since using multilevel analysis showed some differences in metabolites at baseline Figure 2A, Day-1 and Figure 3A, Day-1), we used multivariate classification models, to compare the metabolic differences between Europeans and Africans as well as between TBS positive and TBS negative Africans. The score plot of sPLS-DA model (Figure 4A), showed that Europeans and Africans clustered separately. Three of the 53 metabolites, namely, trigonelline, imidazole and 1-methylnicotinamide, were top ranking metabolites with the highest variable importance score in the model (Figure 4B) and were significantly higher in Europeans (Figure 2, Day-1 and Figure S1). These metabolites were selected in more than 75% of cross-validation repeats, suggesting reproducibility of the signature (Figure 4C). Figure 4D, showed that the sPLS-DA model segregated TBS positive and TBS negative Africans. Taurine, malonate, pyroglutamate (higher in TBS positive, Figure 4E and Figure S2), and acetoacetate (higher in TBS negative, Figure 4E and Figure S2) were driving the separation (Figure 4F). Overall, these results showed that TBS positive and TBS negative individuals display different metabolite profiles even before challenged with PfSPZ DVI.

### 3.4. Mass Spectrometry Reveals Metabolites Predicting Parasitaemia before PfSPZ DVI

**Figure 5:**
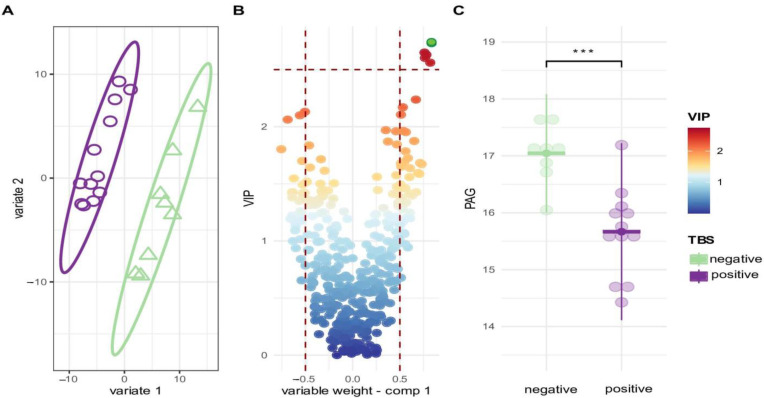
Mass spectrometry reveals metabolites discriminating TBS positive and TBS negative Africans before PfSPZ DVI. (**A**) A score plot of PLS-DA model with a TBS status as a class identity. A classification performance of the model was assessed by five-fold cross-validation with over 1000 repeats; overall classification error rate was 22% for the first and 15% for the second component. (**B**) A plot showing variable weight on the first component (X axis) versus variable importance on projection (VIP) values; the plot reveals a cluster of the features with VIP > 2.5 upregulated in the TBS negative Africans. (**C**) A dot plot showing the difference in a relative abundance between TBS negative and TBS positive Africans for a feature with the highest VIP (i.e., VIP > 2.5, coloured in green). The feature annotated as Phenylacetylglutamine (PAG). *** *p* < 0.001.

**Figure 6:**
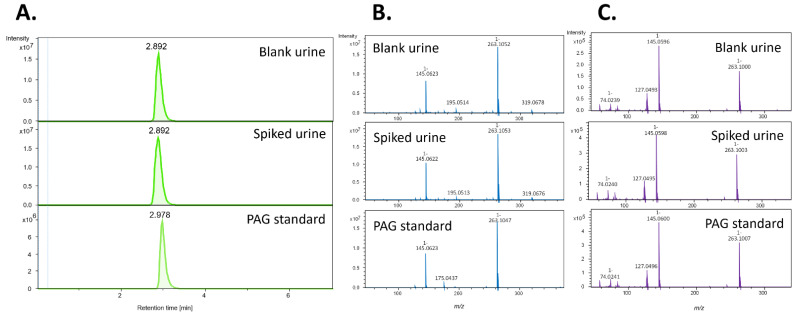
Phenylacetylglutamine may predict parasitemia in Africans before PfSPZ DVI. Confirmation of the identity of phenylacetylglutamine (PAG) with the analysis of a sample composed of pooled urine samples collected from TBS negative Africans at Day-1 before PfSPZ DVI (Blank urine), the same urine sample spiked with PAG (Spiked urine) and a neat standard of PAG (PAG standard). (**A**) Extracted ion chromatogram, (**B**) MS spectrum and (**C**) MS/MS fragmentation spectrum, of PAG in blank urine, spiked urine and PAG standard. The retention times, as well as MS and MS/MS spectra of PAG overlap in the different samples injected, confirming the identity of PAG (confidence level 1 in the metabolite identification). Experimental conditions: see main text.

While NMR is well known for its reproducibility, its analytical sensitivity remains limited, especially compared to mass spectrometry (MS)-based approaches. As the possibility to explore further the potential of identifying metabolites that discriminate between those who become parasitaemic and those that control infection, we applied untargeted MS-based profiling of urinary metabolites. To this end, hydrophilic interaction chromatography-mass spectrometry(HILIC-MS) was used to analyze baseline urine samples. A sPLS-DA modelling of the HILIC-MS data showed a different clustering of urine samples between TBS positive and TBS negative Africans (Figure 5A). Figure 5B identified the most important features of the PLS-DA model and indicated cluster of features with the highest variable importance in projection (VIP > 2.5) scores. These features are related to a metabolite, which we identified as phenylacetylglutamine (PAG). Figure 5C showed that PAG is significantly higher in TBS negative Africans. PAG was first tentatively identified using exact mass, isotopic distribution, MS/MS fragmentation pattern, and comparison with databases. The identity of PAG was confirmed (confidently annotated compound, confidence level 1 in the metabolite identification [23]) with the injection of PAG neat standards, a sample composed of urine samples collected on TBS negative individuals at Day 1 before PfSPZ DVI pooled together, as well as PAG spiked into this pool of urine samples (Figure 6).

## 4. Discussion

In this study, urinary metabolites were analysed before and at several time points after challenging volunteers with malaria parasites by the direct intravenous administration of live, aseptic, cryopreserved *Plasmodium falciparum* sporozoites (PfSPZ DVI). The volunteers consisted of malaria-naïve Europeans and Africans with lifelong exposure to malaria. Upon malaria challenge, a total of 23 out of 53 metabolites annotated by NMR showed differences between Europeans and Africans at any given time point. With multilevel analysis, we found the time course of nine metabolites, namely imidazole, phenylacetate, myo-inositol, fucose, cis-aconitate, acetaminophen, acetoacetate, dimethylglycine and pseudouridine, to be significantly different between the two groups. Most changes were seen on Days 5 and 9 post PfSPZ DVI, more prominently in Europeans. Increased level of metabolites such as myo-inositol have been observed in the context of hyperglycaemia in diabetic patient [24], and change in glucose homeostasis is a common feature of malaria severity [25]. As such the increase level of myo-inositol in malaria-naïve Europeans might be a reflection of altered glucose homeostasis as a consequence of the malaria infection. This might be linked with the rapid development of parasitaemia and symptoms in these malaria-naïve individuals. By contrast, dimethylglycine has been described to boost both the humoral and cell-mediated immune response in humans [26]. In line with this, it was found to be increased in malaria pre-exposed volunteers who generally developed parasitaemia at a later time point and were asymptomatic [19]. Of all metabolites, pseudouridine and myo-inositol, which have been linked to inflammation [27,28], showed the strongest differences between Europeans and Africans.

Within the African group, 16 metabolites were different between those who developed parasitaemia and those who controlled their parasitaemia. Of these, 10 showed differential response over time according to multilevel analysis. Among metabolites that changed after infection, myo-inositol and dimethylglycine were shared in the data comparing Europeans with Africans. Myo-inositol was higher and dimethylglycine was lower in TBS positive and in Europeans, both groups could not control parasitaemia. However, the difference in myo-inositol was seen on Day 2 for the TBS positive Africans but on Day 5 and Day 9 for Europeans. Conversely, for dimethylglycine, the difference was seen on Day 5 for the Europeans but on Day 11 for the TBS positive Africans. It important to realise that in Africans who became TBS positive, did so later than in Europeans, indicating the longer control of parasitaemia in Africans. Although highly speculative, the earlier drop in myo-inositol might be associated with a physiological process that prolonged the control of parasitaemia while the opposite might be true for dimethylglycine.

Interestingly, we also observed that some metabolites were already different at baseline between the groups. With the limited number of metabolites identified by NMR, differences were seen between Europeans and Africans that could reflect differences in lifestyle or dietary intake [29]. However, it was also noted that TBS-positive and TBS-negative Africans had different levels of a few metabolites at baseline. Taurine, pyroglutamate and malonate were more abundant in TBS-positive Africans, whereas acetoacetate was higher in TBS-negative individuals. Of these metabolites, pyroglutamate through supporting CD47 signaling [30] and malonate through suppressing inflammatory macrophages [31] could potentially have immunosuppressive effects and thus increased risk of development of parasitaemia. These metabolites that were different at baseline might have a predictive value for identifying subjects residing in a malaria-endemic area capable of controlling parasitaemia. This could have consequences with respect to understanding the mechanisms underlying immunity to malaria or identifying subjects to participate in vaccination trials. Therefore, a more analytically sensitive method was used to measure features of metabolites at baseline in TBS-positive and TBS-negative groups. HILIC identified phenylacetylglutamine (PAG) to be highly elevated in TBS-negative Africans that could control their parasitaemia. Although this is the first time that PAG is reported in the context of malaria, PAG has been reported to be elevated in urine of *S mansoni*-infected children [32] and in plasma of patients with cardiovascular disease [33]. The opposite association of PAG with malaria versus schistosome might be due to the control of malaria by a Th1 response, while that of schistosome is thought to be associated with a Th2 response.

Several studies have determined the profile of metabolites in malaria, but most were studies of plasma samples of subjects with symptomatic infections [14,16,17,34]. Therefore, our findings cannot be directly compared with previous metabolomics studies. Although the use of CHMI provided a good opportunity to assess metabolic perturbation as the infection develops, like many other studies that investigated metabolomics in malaria, it is also descriptive. Future studies should include not only the validation of these results but also in vitro and in vivo experiments to validate the function of the identified metabolites. Urinary metabolomics profiling in controlled human malaria infection in Gabon was the first study that assessed temporal perturbation of metabolites during malaria infection in two geographically distinct population expressing different level of prior exposure to *P. falciparum*. Our metabolomics analysis highlighted significant differences in urinary metabolite profiles as a consequence of lifelong exposure to malaria and identified metabolites whose changes could be associated with the ability to control parasitaemia and that metabolic wiring might be detectable before challenge.

## Data Availability

The dataset presented in this study are available in supplementary tables.

## Supplementary Materials

The following supporting information can be downloaded at: https://www.mdpi.com/article/10.3390/metabo12121224/s1, Figure S1. Line plots showing the trajectory of change in the concentrations of metabolites in Europeans and Africans with significant *p*-value interaction (*p*-value int < 0.05) effect between group and time. Metabolite concentrations were log2-transformed prior to mixed-model analysis. Error bars represent estimated mean log2 concentration and the corresponding 95% confidence interval. Colors indicate groups. Individual data points are shown. *p*-values of interaction effect were obtained using joint-tests. Figure S2. Line plots showing the trajectory of change in the concentrations of metabolites in TBS positive and TBS negative Africans with significant *p*-value interaction (*p*-value int < 0.05) effect between group and time. Metabolite concentrations were log2-transformed prior to mixed model analysis. Error bars represent estimated mean log2 concentration and the corresponding 95% confidence interval. Colors indicate groups. Individual data points are shown. *p*-values of interaction effect were obtained using joint-tests. Table S1. The CHMI trial volunteers’ characteristics. Table S2. List of metabolites identified and quantified by NMR. Table S3. List of features obtained by HILIC.
