# Supplementary material for: Urinary Metabolic Profiling in Volunteers Undergoing Malaria Challenge in Gabon
Source: Metabolites. 2022 Dec 6;12(12):1224. doi: 10.3390/metabo12121224 (PMC9783708; doi:10.3390/metabo12121224)
Supplement: Supplementary file 1 [file metabolites-12-01224-s001.zip › Figure S1.pdf]

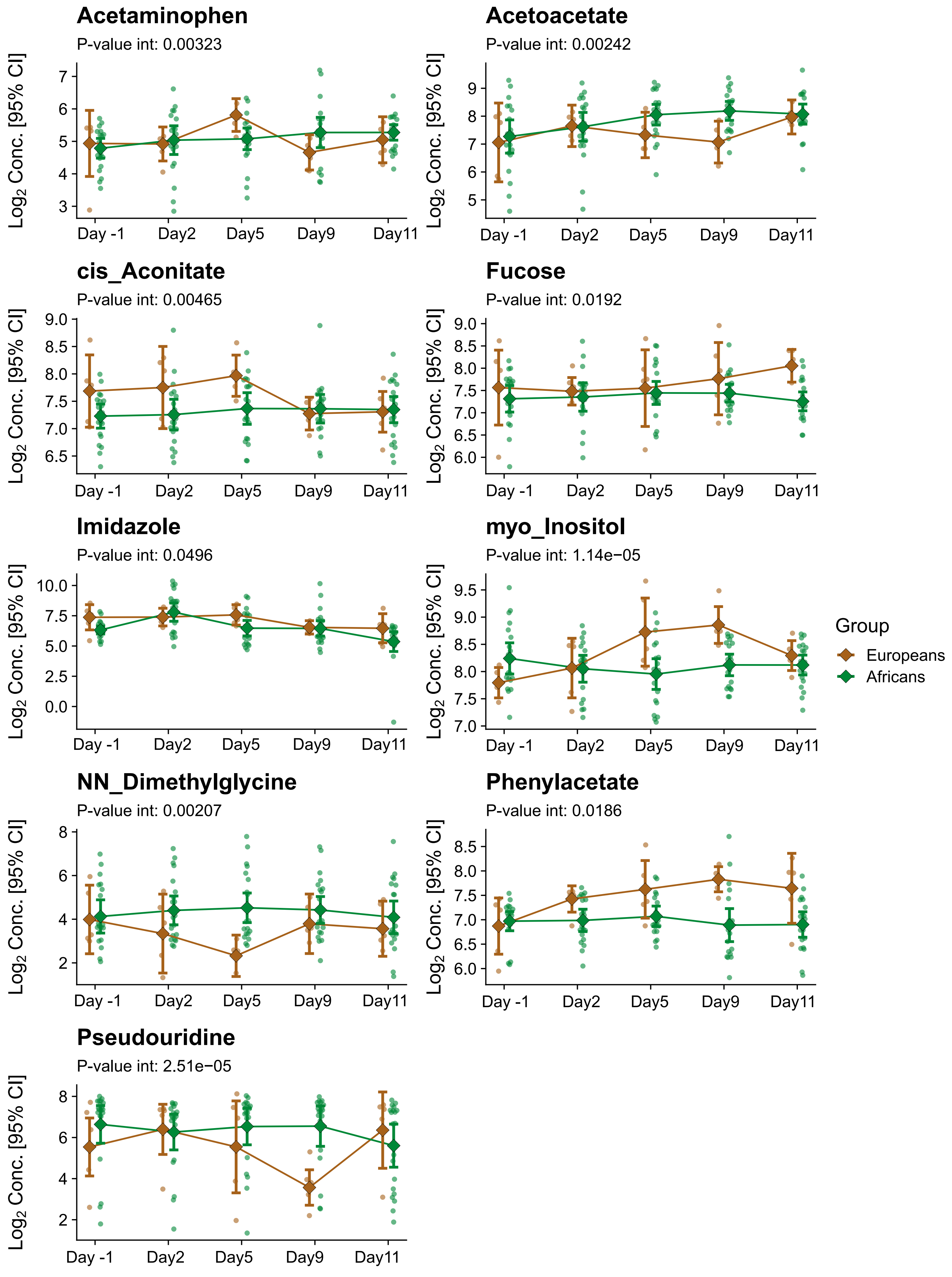
Acetaminophen
P-value int: 0.00323
Acetoacetate
P-value int: 0.00242
cis_Aconitate
P-value int: 0.00465
Fucose
P-value int: 0.0192
Imidazole
P-value int: 0.0496
myo_Inositol
P-value int: 1.14e−05
NN_Dimethylglycine
P-value int: 0.00207
Phenylacetate
P-value int: 0.0186
Pseudouridine
P-value int: 2.51e−05
Log2 Conc. [95% CI]
Day -1
Day2
Day5
Day9
Day11
Group
Europeans
Africans
