# Supplementary material for: Urinary Metabolic Profiling in Volunteers Undergoing Malaria Challenge in Gabon
Source: Metabolites. 2022 Dec 6;12(12):1224. doi: 10.3390/metabo12121224 (PMC9783708; doi:10.3390/metabo12121224)
Supplement: Supplementary file 1 [file metabolites-12-01224-s001.zip › Table S1.pdf]

Table S1. The CHMI trial volunteers' characteristics.

Characteristic	Europeans	Africans	
		TBS positive	TBS negative
n	5	12	8
Sex, male (n, %)	1 (20%)	8 (66.7%)	4 (50%)
HbAA haemoglobin phenotype	5 (100%)	7 (58.3%)	4 (50%)
Age, years	28.0 (24.3-28.8)	22.4 (19.0-25.9)	21.6 (18.6-26.6)
BMI, kg/m^2	23.3 (18.9-26.6)	22.6 (16.2-27.0)	19.9 (19.2-22.9)
Parasitaemic by TBS (n, %)	5 (100%)	12 (100%)	0 (0%)
Days to parasitaemia by TBS (GM, range)	12.4 (12-14)	17.9 (13-25)	-

All values are number (%) or median (range) unless mentioned otherwise. All volunteers were treated on Day 28. TBS, Thick blood smear. GM, geometric mean.
