# Supplementary material for: Urinary Metabolic Profiling in Volunteers Undergoing Malaria Challenge in Gabon
Source: Metabolites. 2022 Dec 6;12(12):1224. doi: 10.3390/metabo12121224 (PMC9783708; doi:10.3390/metabo12121224)
Supplement: Supplementary file 1 [file metabolites-12-01224-s001.zip › Table S2.pdf]

Table S2: List of metabolites identified and quantified by NMR

A total of 53 metabolites were identified and quantified using the targeted NMR approach and the concentration (mM) is shown as median, first (q1) and third (q3) quartile .

metabolite	median	q1	q3
Acetaminophen	36.6	22.9	52.4
Acetate	49.1	30.7	75.9
Acetoacetate	229.1	135.6	406.7
Acetone	9.0	6.4	12.7
Acetylglycine	1,075.4	784.2	1,805.3
Alanine	233.6	173.1	320.5
Betaine	87.2	47.7	137.5
Choline	57.1	29.6	95.8
Cis-Aconitate	170.3	116.7	241.9
Citrate	1,119.1	439.1	2,332.3
Creatine	191.8	102.1	379.7
Creatinine	12,994.3	9,006.8	18,180.9
Desaminotyrosine	127.1	93.0	204.1
Dimethylamine	501.9	357.2	658.7
Ethanol	39.4	39.4	39.4
Formate	179.8	128.5	251.7
Fucose	181.6	130.0	265.1
Fumarate	5.1	2.7	8.5
Glucose	425.3	311.2	593.3
Glycine	1,608.4	943.3	2,538.6
Glycolate	722.4	412.2	1,052.7
Guanidoacetate	389.0	222.4	658.1
Hippurate	231.4	125.8	516.4
Histidine	292.5	210.4	455.6
Imidazole	93.5	56.9	171.6
Isobutyrate	35.4	26.1	48.6

metabolite	median	q1	q3
Lactate	67.0	55.4	97.8
Malonate	404.7	303.2	530.7
Methanol	161.1	99.8	307.3
Methylguanidine	220.5	137.7	331.3
Myo-Inositol	261.6	197.4	407.0
1-Methylhydantoin	130.4	99.2	182.5
1-Methylnicotinamide	39.9	24.4	65.6
3-Hydroxyisovalerate	67.4	44.9	97.5
3-Indoxylsulfate	413.6	211.6	721.2
N6-Acetyl-L-lysine	1,669.6	1,219.7	2,299.6
Dimethylglycine	15.2	8.6	50.2
Phenylacetate	141.4	97.8	205.6
3-Methylhistidine	399.7	223.6	616.2
Pseudouridine	133.1	25.2	200.4
Pyroglutamate	253.6	178.3	338.0
Pyruvate	33.0	21.4	50.4
Succinate	36.3	21.1	65.9
Tartrate	19.0	15.1	32.7
Taurine	1,044.2	512.0	1,607.7
Threonine	161.1	115.2	226.9
Trigonelline	29.1	16.4	64.9
Trimethylamine	19.3	13.1	26.1
Trimethylamine oxide	324.4	152.9	624.8
Tryptophan	75.8	56.9	101.1
Tyrosine	132.4	66.4	198.6
Urea	10,314.9	7,933.0	12,800.5
Valine	35.5	29.7	47.9
