# Supplementary figures and images for: Urinary Metabolic Profiling in Volunteers Undergoing Malaria Challenge in Gabon
Source: Metabolites. 2022 Dec 6;12(12):1224. doi: 10.3390/metabo12121224 (PMC9783708; doi:10.3390/metabo12121224)

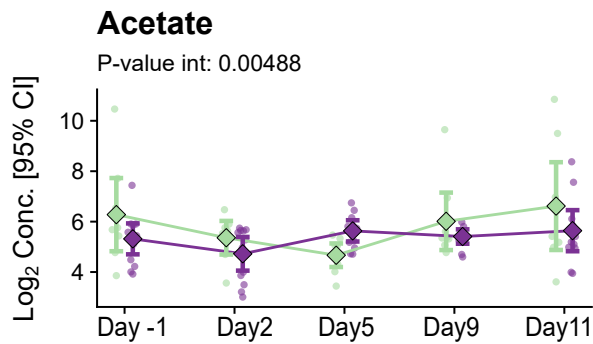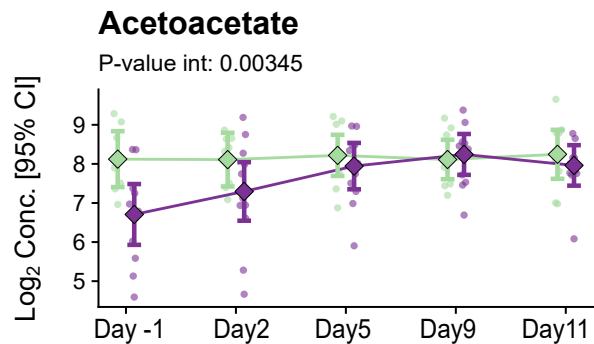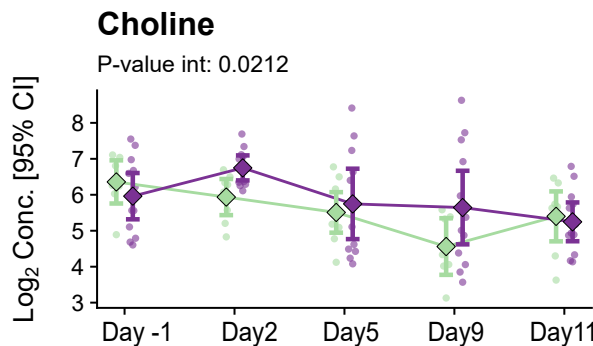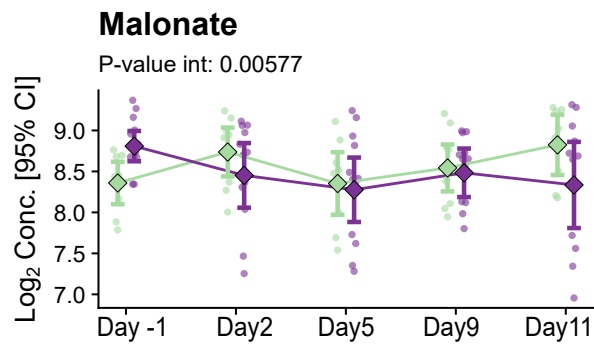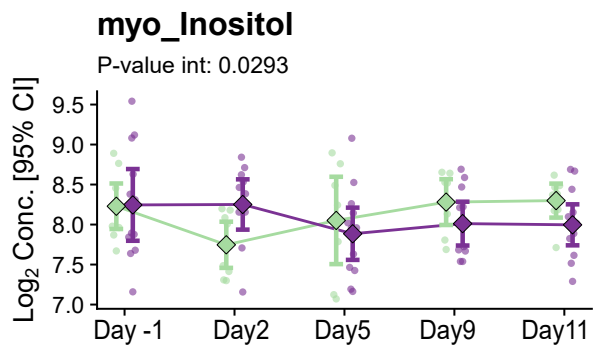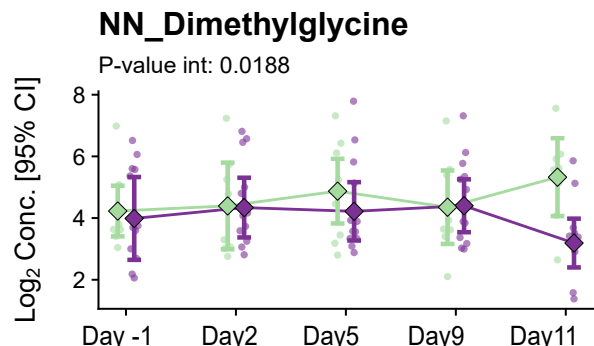

TBS

- ◆ TBS positive
- ◆ TBS negative

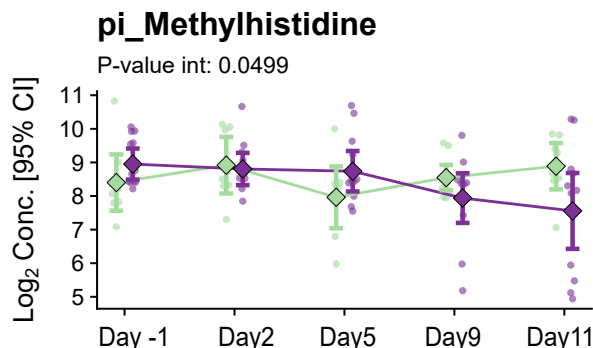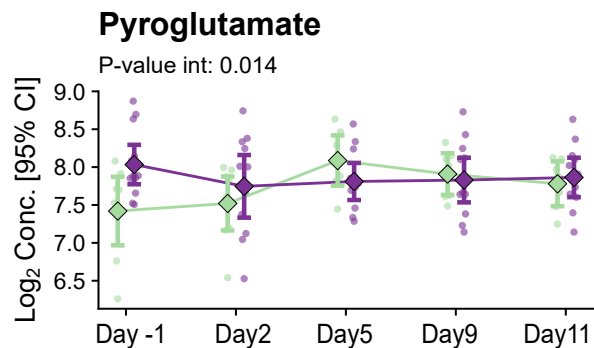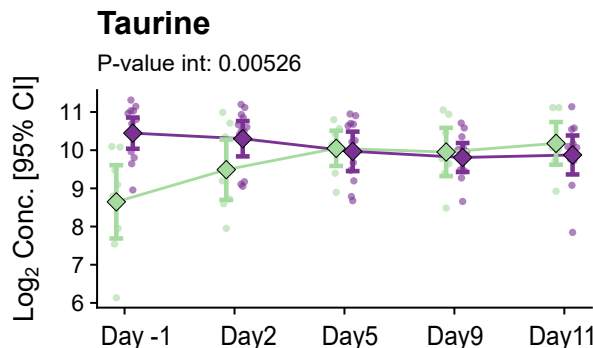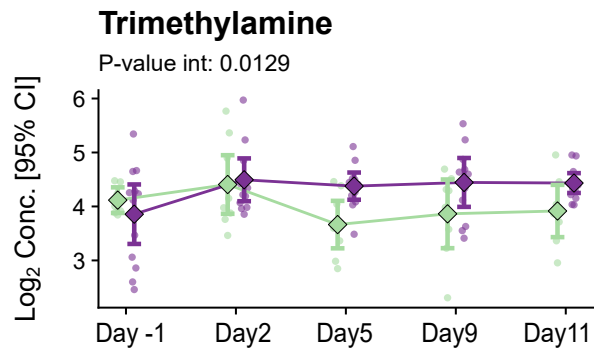

Supplement: Supplementary file 1 [file metabolites-12-01224-s001.zip › Figure S2.pdf]
